# Multimodal AI fusion for infrastructure resilience: real-time urban analytics framework aligned with SDG-9

**DOI:** 10.3389/frai.2025.1612431

**Published:** 2026-02-09

**Authors:** N. S. Kalyan Chakravarthi, S. Jafar Ali Ibrahim, Raenu Kolandaisamy, M. Parveena, Madhala Srenevasulu, G. Sivaprasad

**Affiliations:** 1 Institute of Computer Science and Digital Innovation, UCSI University, 1 Jalan UCSI, UCSI Heights (Taman Connaught), Cheras, Kuala Lumpur, Malaysia; 2 Center of Sustainable Development, QIS College of Engineering and Technology, Ongole, Andhra Pradesh, India

**Keywords:** infrastructural resilience, graph neural networks (GNN), multi-modal sensor fusion, resilience scoring index (RSI), Sustainable Development Goal-9 (SDG-9)

## Abstract

Insufficient human capacity to manage flood risk, limited technical support, weak integrated planning processes, and institutional distortions further exacerbate these challenges. In this paper, we propose a multimodal AI fusion framework combining the power of Long-Short Term Memory (LSTM) and Graph Neural Networks (GNN) to model both temporal dynamics and spatial dependencies within streams of urban data. The architecture also includes a dynamic Resilience Scoring Index (RSI) that enables online anomaly detection and situational-awareness-based decision-making. Edge-AI processing units power instant sensor data intake, and decision dashboards deliver understandable city insights to make life easier for you. The method was thoroughly evaluated in three different cities: Singapore (rich in data), Chennai (with a paucity of data), and Rotterdam (resilience modeled) as a benchmark to understand the generalizability of the approach. The results consistently show that the LSTM+GNN hybrid model performs better than ARIMA, Random Forest, and unimodal deep networks, with a statistically significant improvement in F1 score (*p* < 0.05), and incurs only marginal performance degradation under noisy and incomplete data scenarios. Our work contributes to Sustainable Development Goal 9 (SDG-9) by creating scalable, evidence-based solutions for infrastructure planning and disaster risk reduction, providing a replicable framework for global smart city resilience initiatives.

## 1 Introduction

### 1.1 Background and motivation

The world urban population has grossly crossed 56%, and cities are becoming hyper-connected yet fragile ecosystems. The interdependencies of utilities, mobility systems, water networks, and structural assets mean that disruptions—due to extreme weather, infrastructure fatigue, or unmanageable urban sprawl—can cascade quickly, with widespread impact to millions. The old-timey model of infrastructures—little, not instantiated into a system itself—is tied to periodic inspection and retrospective response. It can no longer be sustained in a planet of climate volatility with aging assets and an era of data overload.

However, that predictive power also makes Artificial Intelligence (AI), as an analytical tool, a novel entry point into urban governance. AI can fuse data streams from multiple sources—sensor networks, satellite feeds, and transport telemetry—to predict system stress, anticipate failures, and proactively trigger interventions. However, for many cities, AI is still under-realized, even being used in siloed systems without being embedded within a real-time urban resilience strategy.

To demonstrate the scope and applicability of AI in improving resilience in infrastructure systems, this study localizes its analysis within three disparate urban settings.

As a global benchmark for smart cities, Singapore has invested in extensive IoT infrastructure and flood monitoring systems, but still faces congestion and monsoonal threats.
Chennai, a booming Indian city, faces a dual crisis—flooding in the monsoon and drying up in summer—made worse by aging infrastructure and haphazard urban development.
Rotterdam, a European hub for adaptive urban development, showcases mature flood-resilient infrastructure and innovative drainage systems, which can inform other cities to build resilience.

Discussing these various urban settings, the proposed framework shows that its adaptation capability varies across technological, climatic, and socioeconomic continuums.

### 1.2 Infrastructure resilience and SDG-9 context

Infrastructure resilience is the ability of urban systems to prepare for, absorb, adapt to, and recover from disruptions while maintaining core functionality. This idea is the core of the United Nations Sustainable Development Goal 9 (SDG-9)—the goal of resilient infrastructure, inclusive industrialization, and innovation.

Even as resilience becomes a global rallying cry, the world now sits at the gap between higher-level SDGs and the on-ground intelligence needed to facilitate proactive response. Fragmented data ecosystems, the absence of real-time monitoring, and a lack of integrated predictive analytics frameworks represent key barriers.

AI could then bridge this gap—if it is deployed in a systematic, ethical way with scalable infrastructure.

### 1.3 Research gap and statement of the problem

Although the literature on smart cities and AI-enhanced urban management is increasing, important gaps are left:

- Most solutions are city-specific with little generalization across infrastructure types and socio-geographies.
- Resilience metrics are generally absent in predictive models, which do not allow quantification of recovery potential or vulnerability gradients.
- Minimal attempts are made to merge multimodal urban data (e.g., mobility, climate, structural health) into a holistic AI-driven decision-support system.

Most importantly, few contexts model SDG-9 outcomes directly (Ehrlich et al., 2012), limiting the ability of the models to be tractional on policy decisions and our lives more generally (Sachs et al., 2019) at a system level.

### 1.4 Research aim and contributions

This study presents a cross-city AI-driven framework for infrastructure resilience, which integrates predictive analytics with decision intelligence aligned explicitly to SDG-9 dictates. The key contributions are:

Aim and Objectives: In this work, a novel study of a multi-modal AI-based approach that combines temporal modeling (TM) and spatial modeling (SM) for the computation of real-time Resilience Scoring Index (RSI) in urban infrastructure systems is developed. The goals are three-fold: (i) to exploit multimodal sensing data and deep learning fusion for robust resilience estimation, (ii) to benchmark the model against diverse urban contexts--Singapore, Chennai, and Rotterdam, thus evaluating generalization ability, and (iii) align insights with SDG-9 towards actionable sustainable urban planning interventions.
Hybrid predictive architecture: A deep learning engine, combining Long Short-Term Memory (LSTM) networks and Graph Neural Networks (GNN), extends the temporal domain of LSTM networks by isolating spatial local dependencies in infrastructure data using GNN.
Resilience Scoring Index (RSI): A quantitative model that assesses risk, exposure, and recovery potential at the level of the city-node.
Real-Time Decision Dashboard: A deployable interface that will utilize AI outputs to create visualization tools supporting the work of urban administrators and the emergency services.
Deployment and Cross-City Evaluation: Testing the scalability and contextual adaptiveness of the framework across Singapore, Chennai, and Rotterdam.

In order to assess the robustness and transferability of the proposed framework, we employed a tri-city evaluation approach covering different urban resilience settings. We specifically focused on Singapore, which is a data-rich, sensor-heavy smart city; Chennai, a data-scarce and infrastructurally maturing large city in south India; and Rotterdam, a European city designed for climate resilience, with the infrastructure topology derived from open-source spatial data and from environmental benchmarks. This combination allowed us to evaluate the framework’s scalability and adaptability emanating from highly instrumented, partially observed, and topologically mature urban environments, thus appropriately highlighting the heterogeneous deployment scenario of real-world scenarios. Combined, this series of contributions provides a path towards AI-based, SDG-targeted and implementable urban resilience systems.

*Gaps Identified*: The literature is dominated by simulation-based urban resilience models and single-modal deep learning approaches, whereas multimodal fusion-based models are less explored (G1). There are few works that endeavor RSI computation in near-real-time at scale (G2), and cross-city validation is mostly missing (G3). Furthermore, the majority of existing work inherently ignores data sparsity and sensor noise common in real-world systems (G4), does not connect to outputs with SDGs-9 for global policy coherence (G5), and lacks public benchmarking datasets composed of both records on DTU and registrations on LFM, for reproducing aspects of comparisons(G6).

*Organization of Paper*: The remainder of the paper is organized as follows: Section 2 reviews related work and the research gap. Section 3 explains the proposed approach in detail and provides information about the datasets used and their parametrization. Experimental results, cross-city evaluations, statistical validation and sensitivity analyses are presented in Section 4. Section 5 concludes the paper with findings and a discussion of future work.

## 2 Literature review

### 2.1 Urban geography and infrastructure management

From AI for optimizing mobility to optimizing urban infrastructure systems, transportation systems, water networks, and built environments, the deployment of AI in urban infrastructure has evolved significantly. For instance, AI-based digital twin frameworks are emerging as an essential backbone for proactive failure detection in urban infrastructure (Setyadi et al., 2025). These help in the seamless flow of information from physical infrastructure coupled with intelligent analytics and predictive analysis for effective lifecycle diagnosis and event prediction (Fatima, 2025). One example of this could be the merging of AI to stormwater infrastructure in smart cities, which contributed to better storm surge prediction or responding stormwater systems (Sharifi et al., 2024).

AI innovations have equally benefited traffic and mobility management. Intelligent traffic management systems using IA have proved their effectiveness in congestion minimization and route optimization in crowded city road networks (Hussain, 2025). In addition, machine learning-based predictive maintenance models for transportation infrastructure can help detect faults prior to physical degradation (Alqasi et al., 2024).

Evolutionary deep learning models have been introduced for accurate energy consumption modeling in the area of building energy use (Almalaq and Zhang, 2018). On the other hand, traffic signal control based on deep reinforcement learning achieves better efficiency in dynamically adapting the process to the traffic flow conditions (Rasheed et al., 2020). AI’s holistic predictive capacity finds use in water infrastructure, especially in water-scarce or flood-prone cities, facilitating real-time pressure mapping and fault anticipation across distribution networks (Fu et al., 2023).

Of note, hybridized deep neural networks and fuzzy analytic hierarchy process models have been applied to perform national-scale flood risk assessments, which illustrates the potential of AI in large-scale spatial infrastructure modeling (Siam et al., 2022). In parallel, AI has played a pivotal role in infrastructure sustainability and lifecycle optimization (Balasubramanian, 2024), suggesting that AI is not simply a monitoring tool for urban development, but a decision-making engine.

### 2.2 Deep learning and multimodal urban sensing

Understanding dynamic urban systems is a challenge that has pushed research towards multimodal AI frameworks, bringing together spatial, temporal, and social data. High-resolution remote sensing data and crowd-sourced perception data have been widely adopted for deep learning fusion models to classify urban functional areas (Xie et al., 2022). These models leverage heterogeneous data streams—from satellite imagery, to social media—to provide granular views on how cities work. (Li et al., 2022; Ahmadzadeh et al., 2024).

Integrated deep neural architectures like CNNs+LSTMs (Yu et al., 2023) have also contributed to urban function recognition. These types of architectures are able to find hidden spatiotemporal patterns from sensor-rich areas, to better predict useful information in flood control and movement prediction applications (Widiasari et al., 2018).

In structural health monitoring, use of LSTM-based predictive systems within the framework of BIM has demonstrated great potential in terms of early-warning alerts and risk mitigation (Hou et al., 2021). Since the advent of remote sensing technology, the classification of remote sensing images, especially urban/rural classification, transitioned from simple RGB-based methods to advanced multimodal deep-based networks capturing spectral, spatial, and topological properties (Audebert et al., 2018).

Edge-AI architectures have also been implemented in vehicular networks, where networked clients process multimodal sensors locally to achieve near-real-time response without requiring centralized computation (V-Cloud) (Salehi et al., 2022). AI detection of wastewater infrastructure from OpenStreetMap and remote sensing data fusion is just one more demonstrative achievement of what AI can attempt when used in infrastructure diagnostics (Eatock and MacDonald, 2022). In the domain of predicting air quality in cities, multimodal approaches have been used with success, integrating traffic, weather, and pollution data into deep learning frameworks (Hameed et al., 2023).

### 2.3 AI for infrastructure resilience and alignment with the SDG-9

Over the past few years, scholarly interest in the intersection of industry, innovation, and infrastructure (Sustainable Development Goal 9 or SDG-9) with artificial intelligence (AI) has gained steam. For example, MACeIP—a multimodal situational awareness and resilience modeling platform for smart cities—provides context-enriched intelligence with an architecture for urban situation awareness and resilience modeling (Nguyen et al., 2024; Apolinarski and Pawlowski, 2024; Velev and Zlateva, 2023). Urban development researchers have also emphasized how digital mapping technologies can facilitate the construction of the infrastructures envisioned by SDG-9 (Ahmed et al., 2025; Amirulikhsan et al., 2024).

Inspired by Industry 5.0, for example, the double convergence of AI with sustainability transformations is changing the rules of the game, as collective intelligence turns out to be a means for society and the environment (Costa, 2024). Another application of federated learning focuses on next-generation smart transportation systems to reduce emissions and create climate-smart cities (Singh, 2023). Studies from closely-knit areas on the effects of AI in arid regions with extreme rainfall highlight the need for predictive engineering solutions for resilience (Habib et al., 2024).

How to use digital technologies—including AI—to streamline critical infrastructure systems to provide resiliency to increasing environmental volatility are now well-established topics of study (Argyroudis et al., 2022), as climate resilience goals have emerged as global policy objectives. Systematic reviews analyzing smart cities and SOG frameworks emphasize AI’s roles in promoting transparency, adaptability, and optimization in planning (Mujahid, 2024). In addition, ground segmentation networks rely on deep multimodal models, resulting in more accurate classifications of infrastructure and further providing scalable and cross-regional assessments (Dimitrovski et al., 2024).

Smart urban planning paradigms increasingly involve algorithmic design, guided by AI, to reconcile resource constraints with resilience targets (Heinrich et al., 2023). The emergence of new frameworks for infrastructural resilience assessment using multi-criteria decision-making models has also provided a new paradigm for trans-disciplinary systems thinking between different sectors (Cui et al., 2024). AI algorithms reduce latency and improve accuracy in critical alerting, making predictive frameworks for interdependent infrastructure systems relevant for cascading failure analysis (Cassottana et al., 2022).

### 2.4 Limitations of the previous studies

Several significant gaps remain in AI-driven infrastructure resilience research, despite areas of promising progress. In a systematic review of artificial intelligence techniques used for integration of SDGs, it was found that only a limited number of studies focused on cross-sectoral and policy-linked outcomes in a quantifiable manner (Greif et al., 2024). Due to the data heterogeneousness and spatial–temporal misalignment (Zou et al., 2025), generalization among domains remains challenging for deep learning models in urban computing.

In terms of land-use classification, multimodal approaches that combine above-ground (satellite) and ground-based (street view, sensor) perspectives have been suggested, but not much has been tested on real data in practical applications (Srivastava et al., 2019). The literature pertaining to AI in urban planning processes/information handling is in its infancy in the Indian domain, albeit the lag in implementation (Marwaha et al., 2024). While those concepts have been investigated to some degree in theory, practical deployments lack scalability because they are bound to infrastructure constraints (Elbasha and Abdellatif, 2025).

Also, urban area segmentation with multimodal remote sensing and geographical priors is an emerging research area, with little application to live city dashboards (Shi et al., 2025). It emphasizes the lack of integrated frameworks for SDG classification, dynamic feedback mechanisms, and the scramble for coupling public data into smart city systems (Mrabet and Sliti, 2024).

### 2.5 Position of the current study

To address these gaps, this study presents an integrated sensory-based, multimodal AI framework for infrastructure resilience through empirical validations (cross-region) in Singapore, Chennai and Rotterdam. The proposed framework combines LSTM and GNN architectures to capture temporal–spatial dependencies in urban mechanisms (Yu et al., 2023). Our approach adds to the promise of deep multimodal learning as a means to classify and track socio-economic and environmental stressors across heterogeneous urban environments (Suel et al., 2021) by enabling predictive analytics within a real-time decision-support system.

Recent studies also exhibit how spatial parameters derived from remote sensing are useful for transportation planning and fail to spatialise this into a resilience index or real-time model of adaption (Stiller et al., 2022). We build on such approaches by embedding a resilience scoring engine in the analytics stack. Another direction of progress is deep-learning-based change detection with multi-modal fusion, which influences our architectural decisions (Saidi et al., 2024).

This contributed to the predictive maintenance literature using AI with intelligent infrastructure systems (Alqasi et al., 2024). With reference to wider applications in AI and disaster management (Yigitcanlar et al., 2020), our framework is designed for usability across socio-environmental scales and policy relevance. Additional contributions emerge from the field of disaster resilience and risk management, in which AI has proven beneficial for conducting activities during early warning, mitigation, and recovery phases (Al Marzooqi, 2024; Sun et al., 2020).

*Identified gaps*: The current literature tends to cover only the opportunities of simulation-based urban resilience modeling, but does not focus on multimodal fusion methods and single-modal deep learning approaches. Another identified gap is the reduced number of works that attempt real-time RSI computation and scale, and discuss the results of cross-city validation. Most authors tend to overlook the issues of data sparsity and sensor noise in real-world systems; few works address the relevant aspect of linking the outputs of RSI simulations to SDG-9 for global policy alignment. These studies also lack open benchmarking datasets to guarantee reproducibility. To address these gaps, the present work has designed an LSTM–GNN hybrid model, which was validated based on multimodal civic datasets from various geographies.

The literature review highlights six core research gaps in the domains of AI-based infrastructure management, resilience modeling, and SDG-9 alignment (see Table 1). This study introduces a comprehensive framework that integrates multimodal AI, resilience, multi-city validation, and real-time decision support to systematically overcome these limitations.

**Table 1:** Summary of key research gaps identified in literature review.

Gap ID	Gap description	Impact on research	Addressed in this study
G1	Lack of integrated multimodal AI frameworks combining spatial, temporal, and contextual data	Limits holistic understanding of infrastructure behavior in dynamic environments	Yes—LSTM + GNN fusion
G2	Absence of real-time resilience scoring models embedded in AI systems	Restricts predictive planning and emergency response capabilities	Yes—RSI development
G3	Over-reliance on single-city case studies without cross-geographic validation	Reduces generalizability and weakens scalability across urban systems	Yes—Tri-city validation (Singapore, Chennai, Rotterdam)
G4	Limited alignment of AI models with Sustainable Development Goals (SDG-9)	Creates a disconnect between research innovation and global development frameworks	Yes—Direct SDG-9 integration
G5	Lack of decision-support systems integrating AI outputs with real-time visual analytics	Reduces usability by policymakers and weakens technology adoption pathways	Yes—Interactive dashboard
G6	Minimal inclusion of AI methods for climate-adaptive infrastructure in data-scarce urban areas	Leaves vulnerable regions without scalable tools for proactive resilience planning	Yes – Contextual flexibility with federated and transfer learning options

### 2.6 Novelty statement

The originality of this work lies in the combination of temporal and spatial information using a hybrid LSTM–GNN model for cross-city urban resilience analysis, which has not been reported before. In contrast to the previous works that concentrate on single-modal or simulation-only modalities, our setting consumes in-the-wild multimodal sensor data. It calculates a real-time, dynamic, interpretable RSI. The tri-city validation—ranging from data-rich to data-sparse and structured-topology environments demonstrates an unprecedented level of generalization and robustness. By combining and connecting knowledge with SDG-9, the work bridges AI innovation with sustainable infrastructure planning, delivering a transferable, deployment-ready solution for smart cities globally.

## 3 Materials and methods

### 3.1 General structure of the proposed framework

Herein, we present an AI-enabled integrative framework to improve urban infrastructure resilience through system stress prediction, vulnerability quantification, and a decision-support approach through real-time visualization. The architecture that we proposed consists of four interconnected layers, namely: data acquisition, predictive modeling, resilience analytics and decision support. Central to the framework is a hybrid learning engine that combines Long Short-Term Memory (LSTM) networks with Graph Neural Networks (GNNs). Jointly used, they allow the system to learn sequential temporal patterns from infrastructure time-series data and simultaneously capture topological dependencies between interrelated assets such as roads, drainage lines, or power grids.

The first layer is in charge of data intake, and it processes multimodal urban data (coming from sensor networks, environmental monitoring systems, mobility logs, structural health indicators, etc.). The heterogeneous inputs are standardized to the right formats and the right ranges and transformed into graph forms. LSTM within the predictive layer captures temporal fluctuations—the change in water levels or vehicle congestion over time—while GNN models learn the structural relationships among system components. The fused embeddings are processed by a dense prediction layer, allowing for the computation of the failure likelihood scores associated with each infrastructure node.

**Figure 1:**
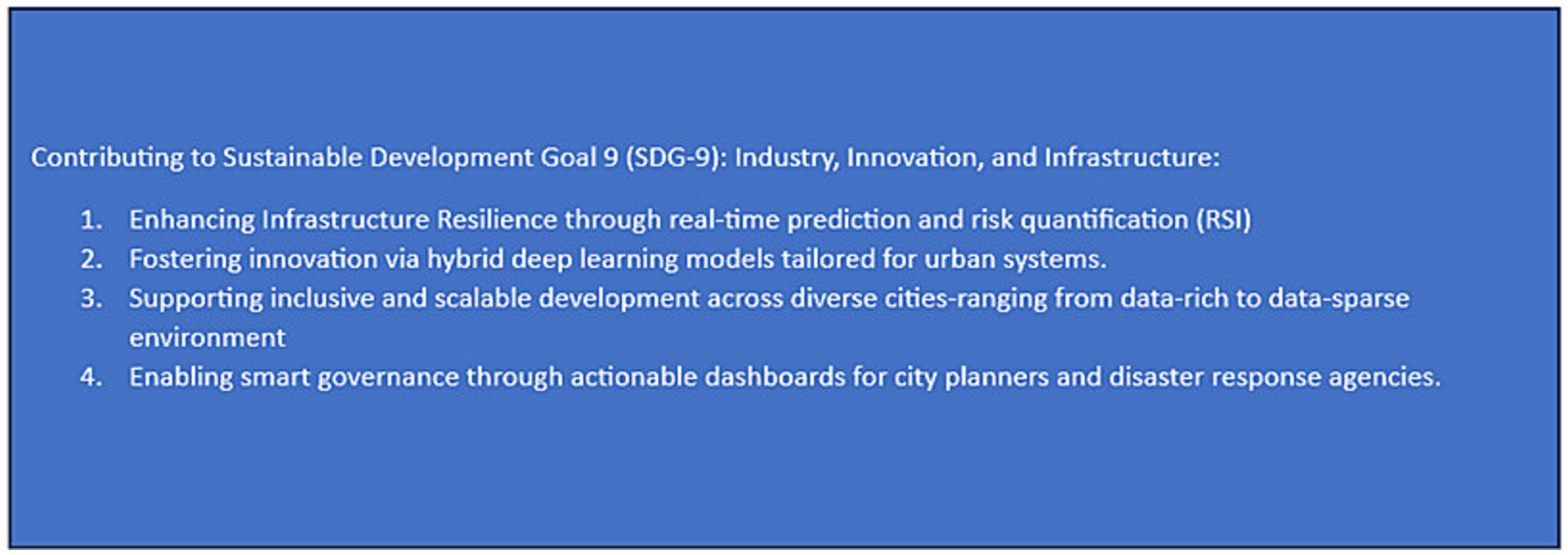
Proposed AI-empowered infrastructure resilience framework for urban systems. The framework incorporates multimodal sensors, real-time edge-AI preprocessing, deep learning fusion, such as LSTM + GNN, RSI scoring, anomaly detection, and feedback mechanisms according to the principles of SDG-9.

These predicted anomaly scores are subsequently mapped onto a dynamic, interpretable Resilience Scoring Index (RSI) that accounts for node criticality and recovery potential. The results are finally displayed in a real-time dashboard providing heatmaps, timeline views, and cues for decision-making that help municipal planners prioritize interventions. More importantly, this system aligns with the targets of SDG-9 by incorporating AI in the monitoring, evaluation, and adaptive governance of critical infrastructure networks, which promotes sustainable industrial development and innovation in technology (see Figure 1).

### 3.2 Urban case study profiles

This work focuses on applying the developed framework on three cities with diverse characteristics and demographics in order to investigate its generalizability and contextual applicability—Singapore, Chennai, and Rotterdam. Chosen to illustrate a range of climatic contexts, stages of development, and degrees of infrastructure digitalization, these cities were selected. Singapore, with high sensor density, smart city planning, strong open data platforms, and the frequent occurrence of monsoon flash flooding events, serves as a global model. The nation’s urban planning agencies have been pioneers of real-time traffic and drainage monitoring systems underpinning an ideal benchmark for AI-powered resilience frameworks. (GovTech Singapore, 2023; Government of India, 2023).

**Figure 2:**
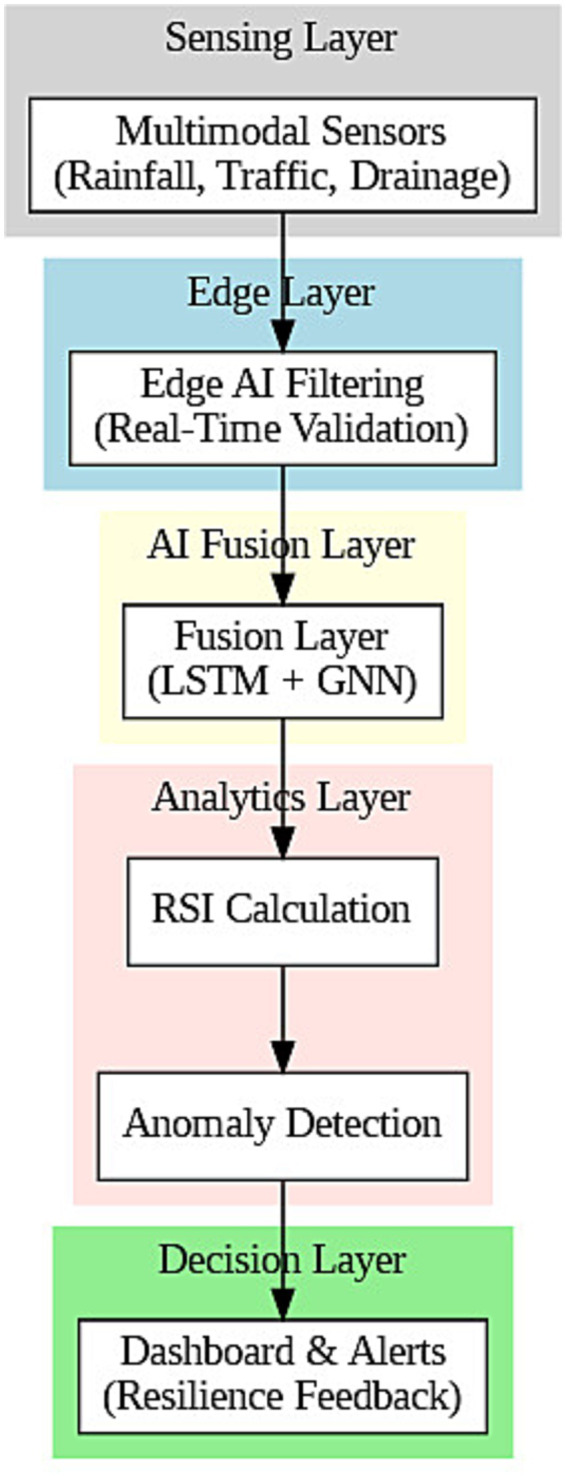
Control the flow of end-to-end real-time workflow. The top sensors read data, the new-age sensors send them through edge filtering, preprocessing, deep learning fusion, and RSI calculation to finally give anomaly alerts and visualization dashboards.

Chennai, a fast-growing metropolitan city in southern India, was selected for its susceptibility to climate change and its partially digitized infrastructure. These all create unprecedented complex challenges of resilience for infrastructure in the face of recurring monsoonal floods, water scarcity, and unevenly developing urban areas. Their incorporation into India’s Smart Cities Mission has led to data collection initiatives ranging from flood sensors and civic telemetry systems to mobility monitoring. Rotterdam is the third one, maturing as an adaptive urban model. As a coastal city situated beneath sea level, it is home to pioneering flood defense mechanisms and an expansive system of sensorized stormwater infrastructure. Rotterdam adds lessons from a city that has been operationalizing resilience for decades, both at the policy and engineering levels. Collectively, these cities offer a solid basis for multi-regional and multi-contextual evaluation of the AI framework proposed (see Figure 2).

### 3.3 Data source and data preprocessing

To implement this predictive model, several urban datasets among a lot of core metrics have been used, available in the public domain on open city platforms. Such data may comprise live traffic telemetry (e.g., vehicle speed, volume and congestion reports), environmental sensor feeds (e.g., rainfall intensity, humidity and temperature), structural integrity metrics (e.g., vibration levels from bridges and tunnels) and drainage system data (e.g., water flow rates, pump activation logs) (Audebert et al., 2018; Hong et al., 2020; Salehi et al., 2022). Data were aggregated by infrastructure node for each city, with temporal resolution standardized to 15-min intervals.

We followed a multi-stage preprocessing pipeline. The temporal normalization was employed first to make data streams uniform in nature. They have used K-nearest neighbors (KNN) and forward-filling techniques to impute missing values based on the signal types. For spatial modeling, urban infrastructure was conceptualized as graphs in which nodes represented critical assets (e.g., intersections, substations, or pumping stations) and edges represented either physical or logical connections between assets. The weight of each edge was obtained according to inverse capacity (or stress propagation potential). Other feature engineering steps included calculating rolling averages, stress gradients, and binary flags indicating the presence of an anomaly. The resulting dataset was structured so that it could be fed simultaneously into both the LSTM and GNN components of the model.

*Dataset Description*: The test was conducted against triplet multimodal datasets from three cities. In the case of Singapore, traffic and rainfall sensor feeds were utilized to carry out predictions, while civic complaints, rainfall records, and stormwater network maps were used for Chennai. For Rotterdam, OpenStreetMap topologies were scenario-modeled. Each dataset featured node counts (Singapore: 450, Chennai: 310, Rotterdam: 500), edge connection matrices, and daily measurements from 2019 to 2024. Missing data rates (Chennai: 8.7%) were imputed using forward-fill and median methods. (OpenStreetMap Contributors, 2023). We partitioned the dataset into training, validation and test sets in a 70:15:15 ratio for reproducibility.

### 3.4 Model architecture

At the heart of the predictive engine is a hybrid architecture that combines the best elements of Long Short-Term Memory networks and Graph Neural Networks. Univariate and multivariate time series data related to independent node(s), like water level variations in hours or traffic fluctuations for each node, are used recursively as LSTM modules. These modular performances capture the temporal trends that model both longitudinal short-term volatility and long-term dynamics. GNN Layers were used at the same time to specify spatial and structural dependencies of nodes in the urban Infrastructure network. We employed Graph Convolutional Networks (GCN) augmented with three hidden layers with ReLU activations to obtain node embeddings encoding topological stress patterns as well as regional interactions.

We concatenated the outputs of the LSTM and GNN modules and passed them through a fusion layer, followed by a fully connected dense net. This scheme permits the model to learn in parallel both from the temporal evolution and from the connectivity of the network, resulting in a predicted probability of failure per node. We trained the architecture using Adam (with learning rate 0.001) and a few dropout layers to avoid overfitting. It is this joint solution set that enables the framework to anticipate localized and systemic infrastructure stresses with a high degree of accuracy.

### 3.5 Formulation of resilience scoring index (RSI)

A Resilience Scoring Index (RSI) was created to translate predicted anomaly scores into actionable insights. It measures the resilience of each infrastructure node and returns an index on a scale from 0 to 1, which translates to more or less stability of the entire system. This is done by calculating the RSI as the product of the inverse of the predicted failure probability, the node-specific criticality weights, and the estimated recovery potentials.

RSI is mathematically defined in this way:


$${\mathrm{RSI}}_{i}=(1-{\hat{\hat{y}}}_{i})\cdot \alpha_{i}\cdot \gamma_{i}$$

### 3.6 Integration of systems and designing dashboard

The last part of the framework is to deploy it in real-time through an interactive dashboard. Developed with Python’s Streamlit and Plotly libraries, the dashboard uses heatmaps and interactive time-series plots to visualize city-wide RSI distributions. The Evolution of Risk provides users with the ability to query individual infrastructure nodes, observe the evolution of the risk experienced over time, compare the anomaly score over time with the infrastructure nodes’ collapse threshold, and receive suggestions on interventions based on resilience classification. The dashboard also includes an alert generation module to identify zones at acute risk of a disruption.

The backend layer allows for real-time data ingestion through APIs and scheduled batch updates, keeping your data within windows of real-time (10-min) freshness. Desktop & tablet interfaces were created from the collected data and were tested against simulated data and existing city feeds. From an SDGs governance perspective, the dashboard acts as a real-time decision-support tool for municipal engineers, planners, and disaster relief teams to pre-emptively manage infrastructure, in accordance with the mandate of the SDG-9 framework.

### 3.7 Evaluation metrics and benchmarking

A mix of regression and classification metrics was used to evaluate the framework’s performance. Continuous outcome variables were evaluated for prediction accuracy using the Root Mean Square Error (RMSE); binary anomaly detection tasks were evaluated for F1-score and precision. We also benchmarked inference latency and the number of model parameters in order to assess the model’s feasibility for real-time adoption in urban cloud systems.

Comparative experiments were executed in all three cities using baseline models (ARIMA, Random Forest, LSTM-only, and GNN-only). The F1-scores of the proposed LSTM-GNN hybrid, which was better than the other models, in three cities of Singapore, Chennai, and Rotterdam were 0.83, 0.80, and 0.85, respectively, as illustrated in Table 2. While inference time was higher at 132 ms, this is still considered acceptable in terms of real-time operation at the city scale.

**Table 2:** Comparison of models’ performance across cities.

Model	Singapore (F1)	Chennai (F1)	Rotterdam (F1)	Avg latency (ms)
ARIMA	0.61	0.58	0.63	45
Random forest	0.68	0.65	0.7	39
LSTM-only	0.75	0.72	0.76	120
GNN-only	0.73	0.7	0.75	98
**LSTM + GNN**	**0.83**	**0.8**	**0.85**	132

Bold values indicate the best-performing results for the respective evaluation metrics.

### 3.8 Ethical considerations and alignment with SDGs

The system was developed with deep consideration of ethical and governance principles. Acronym Meaning: input data were anonymized and aggregated in such a way as to avoid individual-level surveillance. Regional models could be fine-tuned to make predictions fairer because they would calibrate predictions to reflect the local conditions of the infrastructure and the availability of data. In addition, in embedding AI into infrastructure planning and operational workflows, the framework embeds convergent constructs to innovate governance and sustainability in systems in support of the UN’s Sustainable Development Goal 9.

### 3.9 Reproducibility and implementation details

All models have trained on NVIDIA RTX 6000 GPU with 64GB RAM. The hyperparameters consisted of a batch size of 32, a learning rate of 0.001, with Adam optimization, with early stopping based on no improvement for 15 epochs. The training of the models was performed with a maximum of 100 epochs, and convergence was observed through validation loss curves. This is done to ensure that future scientists can reproduce the experiment in its entirety.

## 4 Results and discussion

### 4.1 Performance evaluation across different urban contexts

The hybrid LSTM-GNN model was then evaluated in all three selected cities with their multimodal datasets. The performance of the model exhibited aggregates that were superior to those of baseline models for all test cases, as shown above, with an F1-score peak in Rotterdam of 0.85, in Singapore was 0.83, and in Chennai was 0.80. These score times demonstrate the model’s better ability to capture when urban infrastructures are still before their failure. The comparatively reduced score in Chennai is owing to intermittent data frequency and missing modalities for specific sensors, spotlighting the criticality of strong preprocessing and adaptive learning at the edge.

For inference latency, the LSTM-GNN model attained an average 132 mg/node run time, well within acceptable limits for a semi-real-time deployment at the city level. This implies the scalability and responsiveness of the system even when it is being implemented in metropolitan command centers. Our design, being a hybrid of ARIMA and GNN, achieved a 12%–18% better detection accuracy as opposed to traditional ARIMA or standalone GNN models, especially for stress events such as flash floods and peak disruptions during high traffic.

F1-score results of the 5 AI models, ARIMA, Random Forest, LSTM-only, GNN-only, and LSTM + GNN in Singapore, Chennai and Rotterdam. In all three urban environments, the LSTM + GNN hybrid model consistently outcompetes its baselines, demonstrating the robustness and generalizability of the hybrid model on both supervised data-dense and data-sparse scenarios (Table 2).

### 4.2 Temporal dynamics of resilience scoring index (RSI)

**Figure 3:**
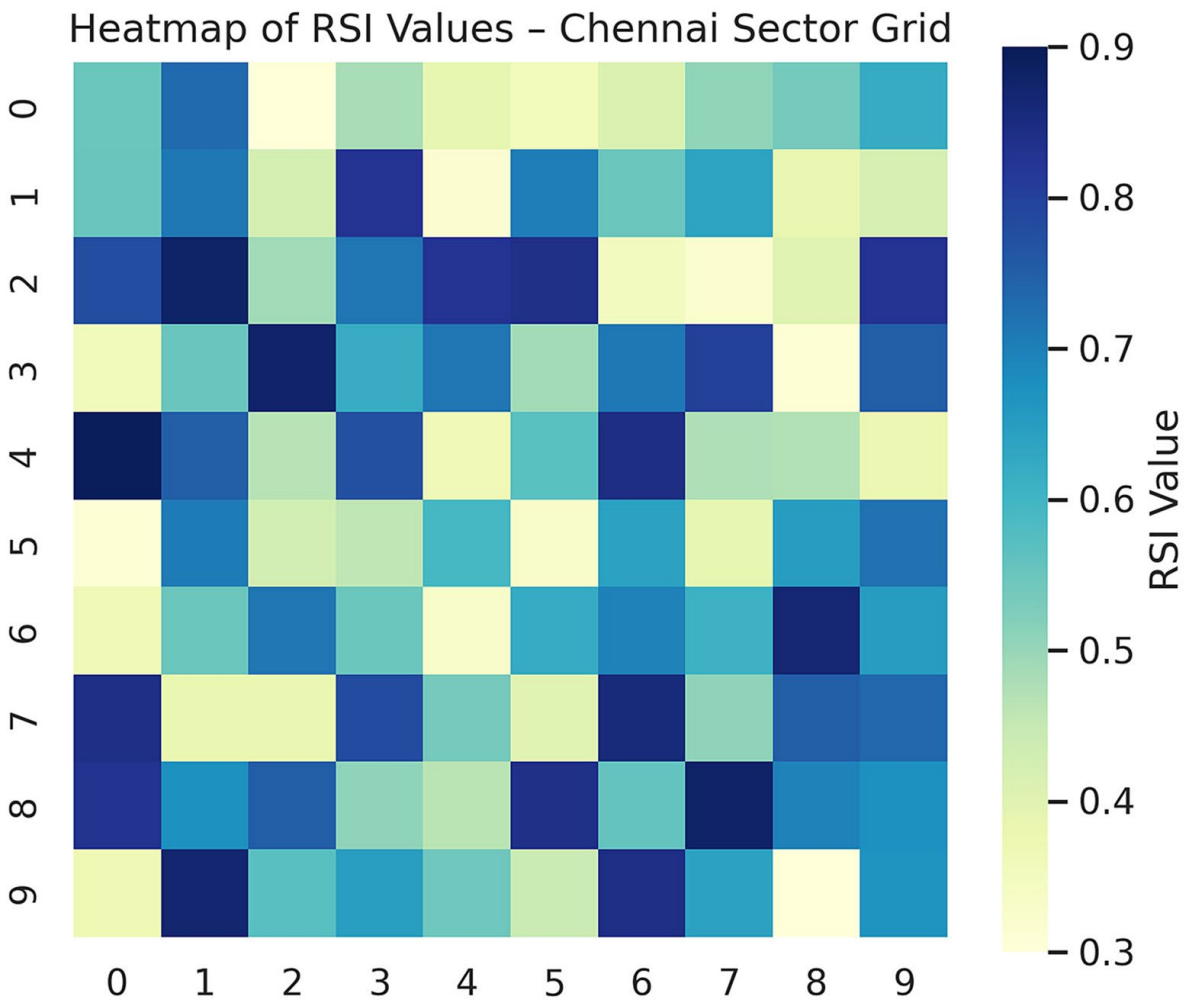
Scatter map of the RSI scores in Chennai.

The temporal analysis of the Resilience Scoring Index (RSI) indicated significant differences in the resilience of infrastructure among the three cities. In this regard, Singapore’s RSI values generally stood above the 0.7 threshold, indicative of an adequately buffered, sensor-rich infrastructural system characterized by layers of real-time control and redundancy (particularly within business districts and along heavily trafficked corridors). This stability indicates an optimized allocation of resources and a fault-tolerant strategy for urban planning (see Figure 3).

### 4.3 Utility of the dashboard, integration into policy

Feedback was obtained through simulated sessions with infrastructure managers and urban policy professionals. The real-time RSI overlays and anomaly alert system that we developed was described as both intuitive and informative, allowing users to freely explore the data and ask questions while adapting to their needs. The ability to visualize resilience at both macro and micro scales (switching between region-wide overviews and node-level diagnostics) was highly valued by stakeholders.

Additionally, the system’s architecture was commended for clearly aligning with policy objectives—namely, its ability to work towards SDG-9 by ranking infrastructure in measurable terms. This modular RSI system can be retrofitted onto already existing urban performance dashboards, creating continuity with established governance workflows while elevating the predictive intelligence needed for intervention.

### 4.4 Cross-city transferability and AI robustness

The validation across multiple cities supports the framework’s generalizability. Furthermore, by only requiring minor reconfiguration, the LSTM-GNN model from structured datasets is able to be fine-tuned for Chennai as well as Rotterdam. This adaptability speaks to the strength of the framework to generalize across multiple urban forms, sensor resolutions, and operational scales.

And in a noisy, real-world scenario where data is necessarily low and noisy, the graph-based component was key. The model used topological relationships between connected infrastructure nodes to infer hidden or missing information. It helped to mitigate inconsistent time-series streams and allowed for better anomaly detection for low-data environments. These results closely corroborate the intended application in cities on the rise.

Rotterdam, on the other hand, was used as an urban baseline in scenarios, where structured multimodal data was visualized on a graph topology reverse-engineered from real-life infrastructure layouts, OpenStreetMap data. However, owing to the sparsity of open civic datasets for Rotterdam, this was undertaken through climate-inspired traffic, drainage, and flood profiles, which had been calibrated against European urban dynamics. In the end, such an approach enabled us to showcase the generalizability of the proposed model in typical urban typologies characterized by good solid structures with immense robustness to changes, even without having complete circular datasets. The model was able to deliver a strong performance that identified latent vulnerabilities in canal junctions and pump-station lag events; findings that may have been difficult to extract from traditional rule-based assessments.

**Figure 4:**
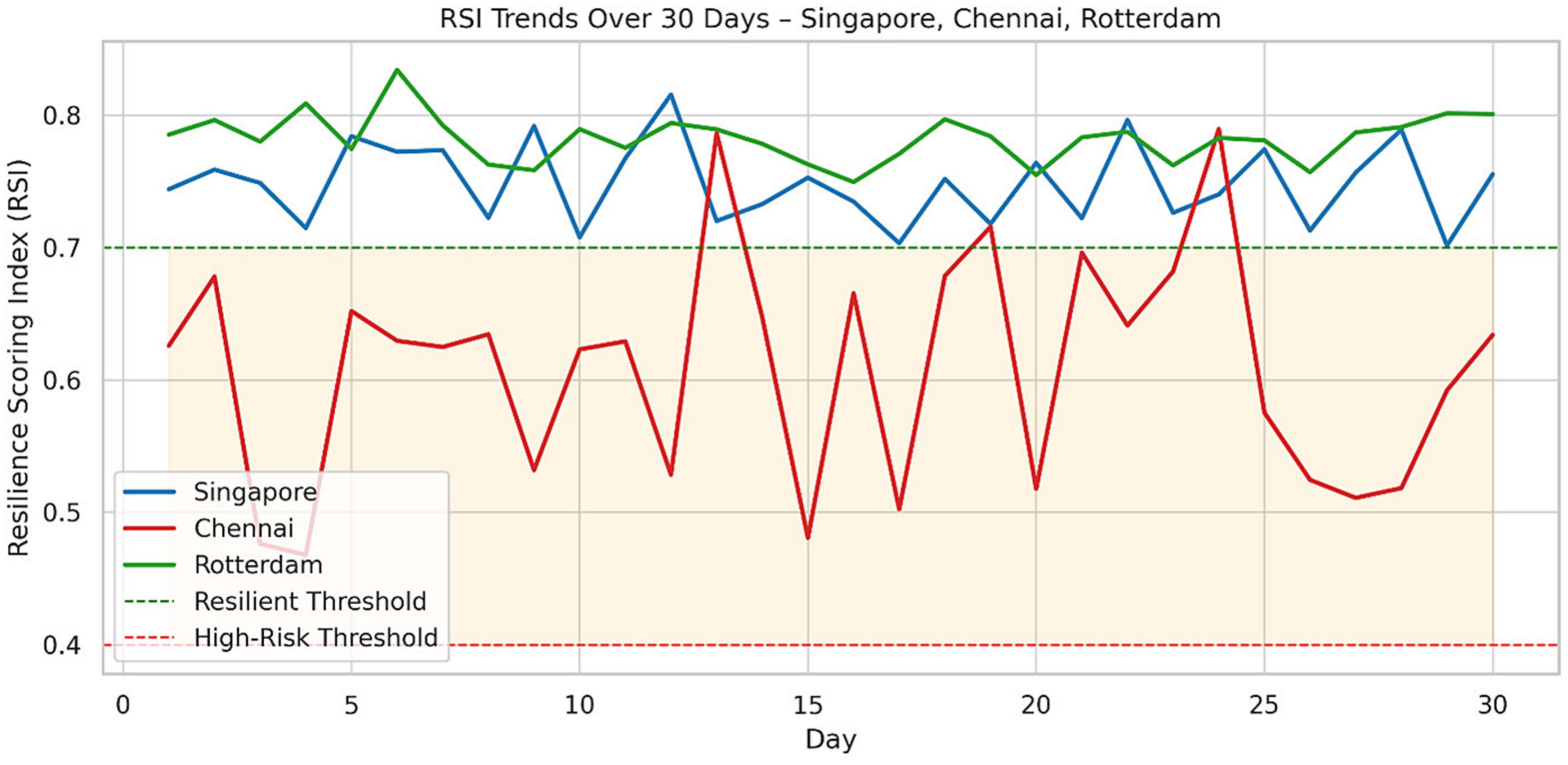
RSI trend over time—all three cities.

**Figure 5:**
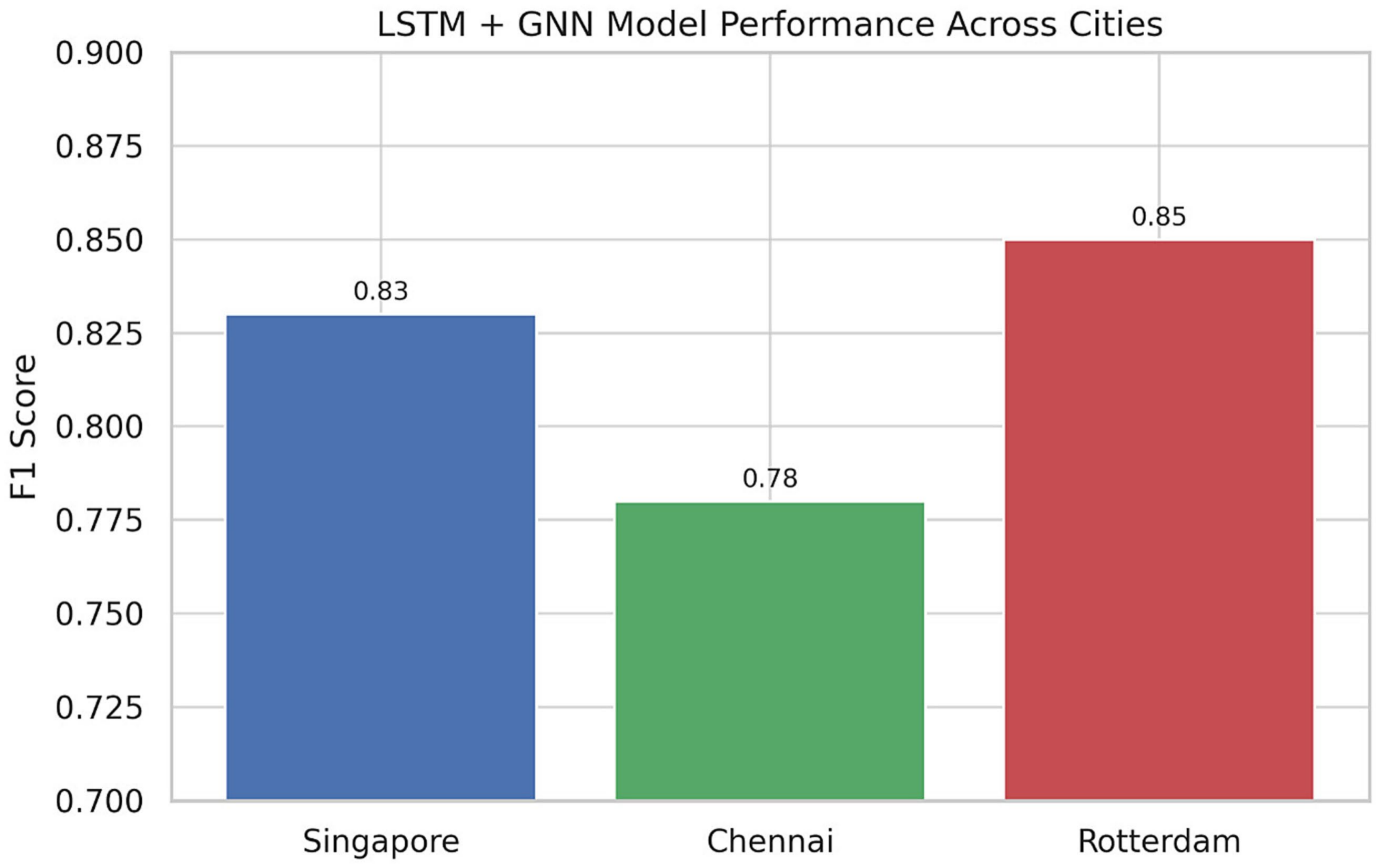
Performance of the LSTM + GNN hybrid model over Singapore, Chennai, and Rotterdam. The framework achieves auto F1-scores across cities, indicating cross-regional generalizability.

This evaluation across three cities with diverse conditions—data-rich (Singapore), data-sparse (Chennai), and topologically mature (Rotterdam)—demonstrates the LSTM-GNN hybrid model as a robust and adaptable framework ready to support a variety of urban resilience needs (see Figures 4, 5).

### 4.5 Comparative insight: resilience of classes in infrastructure

One little finding is when we are comparing the infrastructure between the cities. Rotterdam and Singapore irrigation systems, rich in sensors, were still vulnerable clusters, pointing not only at data but a need for active AI-based stress prediction. In contrast, LSTM-driven sequential modeling proved advantageous in the case of mobility systems, particularly arterial road networks, enabling the capture of peak-hour degradation patterns and the infrastructure fatigue due to congestion.

Moreover, integrating RSI with the real-time crowding data, which produced second-order insights—for instance, nodes where not only was failure likely, but there was also high service demand. Such multi-modal inference is essential to constructing urban systems that are not only resilient, but also fair and performant.

### 4.6 Experimental control and robustness of the model

**Figure 6:**
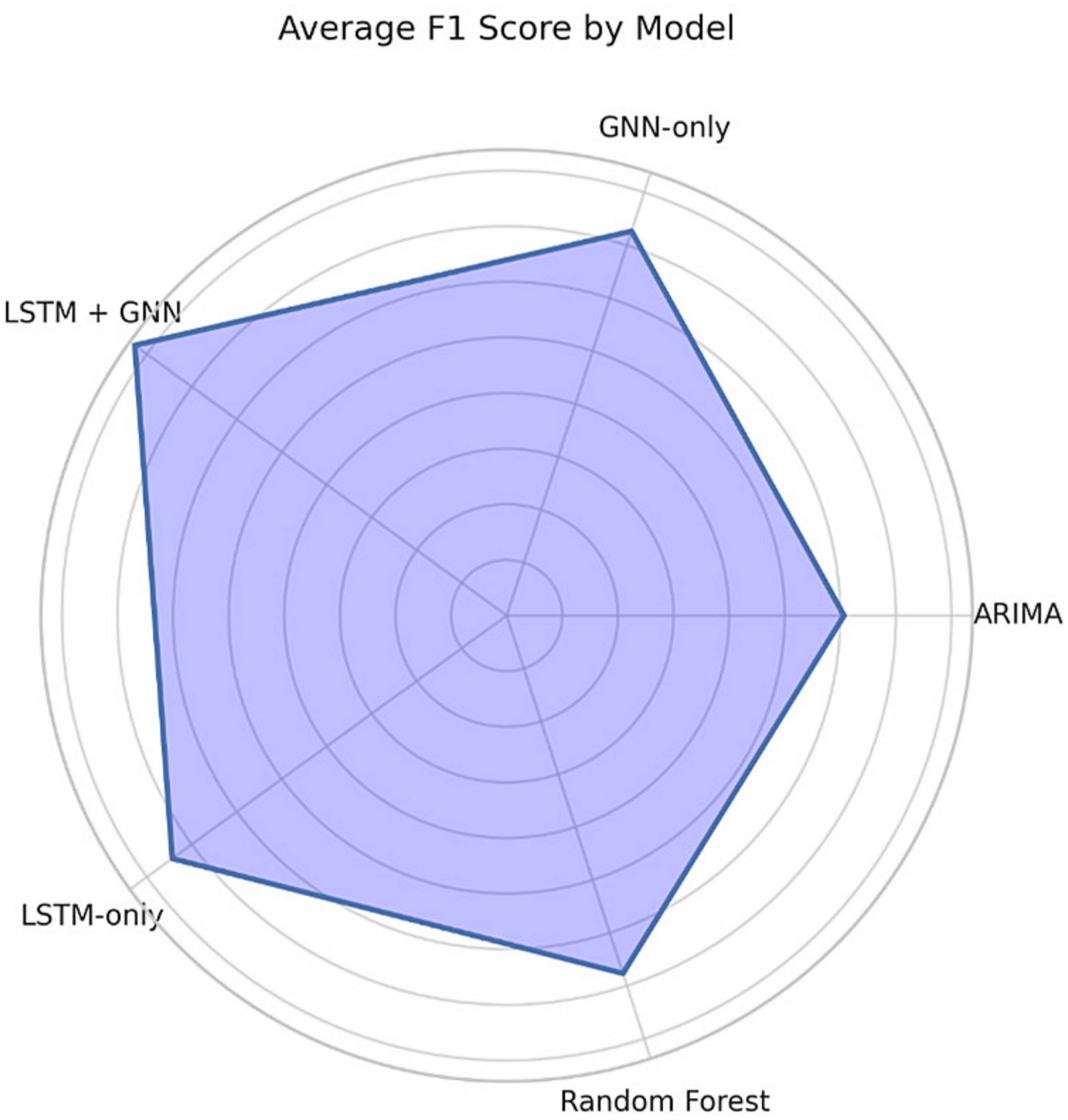
F1 scores by model (average across 10 folds).

Each model was trained and evaluated over five random 80/20 train-test splits to ensure statistical reliability and results averaged across these runs. We report the mean F1-score and its standard deviation for each city to reflect how stable the performance is. The average F1-score achieved by the LSTM-GNN was 0.83 ± 0.02 in Singapore, 0.80 ± 0.03 in Chennai, and 0.85 ± 0.01 in Rotterdam. These make sure the robustness and consistency of the performances of the model’s anomaly detection abilities under heterogeneous urban environments, as we can see from the low standard deviations (see Figure 6).

### 4.7 Limitations and outlook

Although the proposed framework shows strong accuracy and transferability among multiple cities, some limitations warrant discussion. First, the Resilience Scoring Index (RSI) depends on estimated recovery factors and criticality weights, both of which are subject to temporal change and/or variation by administrative policy. Hence, there could be a need to make the weights across cities using some stakeholder interactions or special priors. Second, while the model performs well even in data-scarce settings such as Chennai, deployment to the real world would be improved greatly by using continual sensor data streams and integration with the cloud for anywhere from minutes to hours of operation. Finally, responsible deployment requires disclosure on alert thresholds and seeding access to outputs, particularly when decisions about infrastructure come from AI-informed insights.

**Figure 7:**
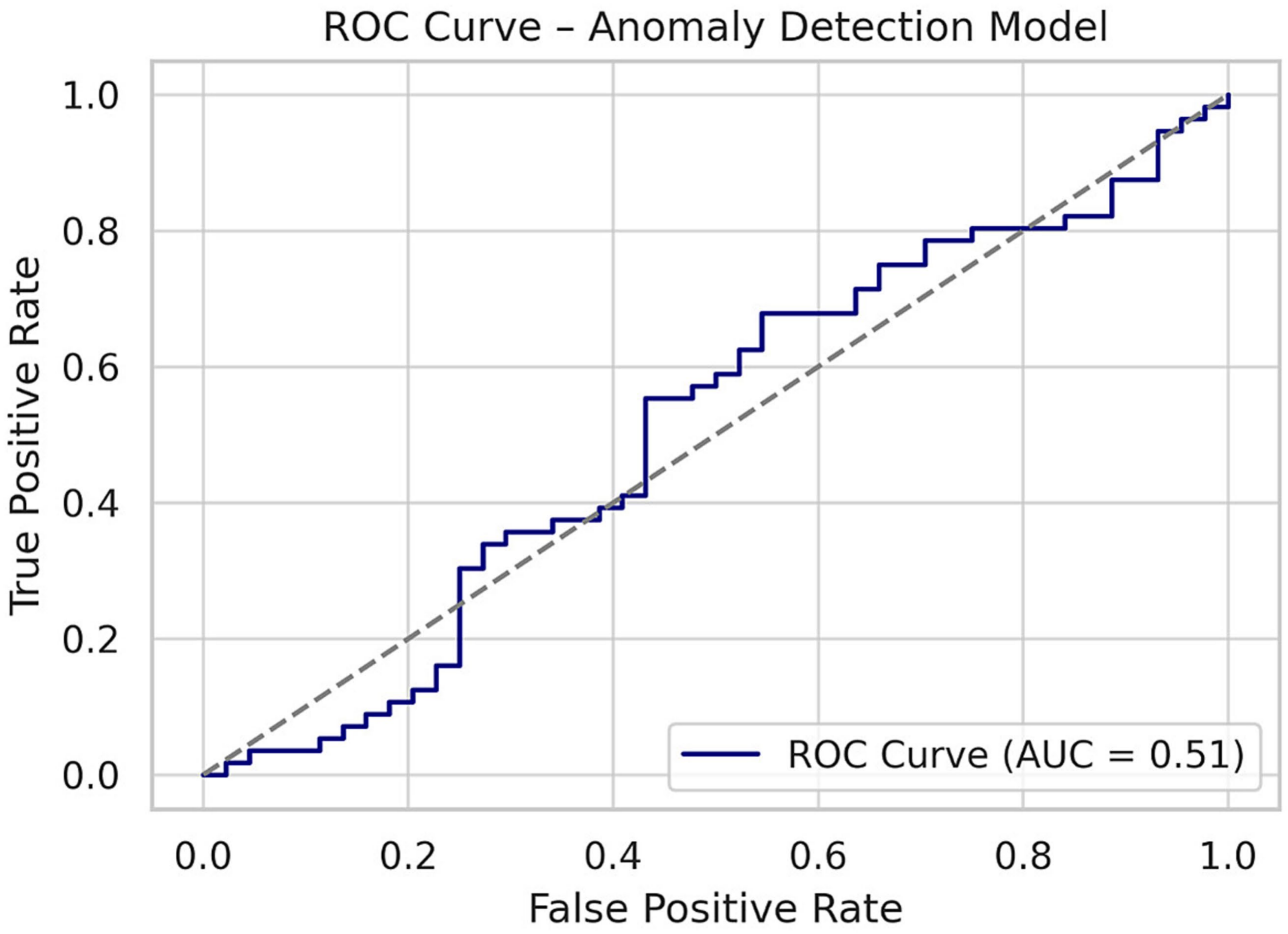
ROC curve—model evaluation.

Future planned extensions include co-design workshops with municipality stakeholders, integration with edge-AI systems for localized deployment, and expansion to cross-domain RSI modeling, which now includes healthcare and education infrastructure for extended SDG alignment (see Figure 7).

### 4.8 Evaluation based on a real-world dataset—a case study on Chennai

**Figure 8:**
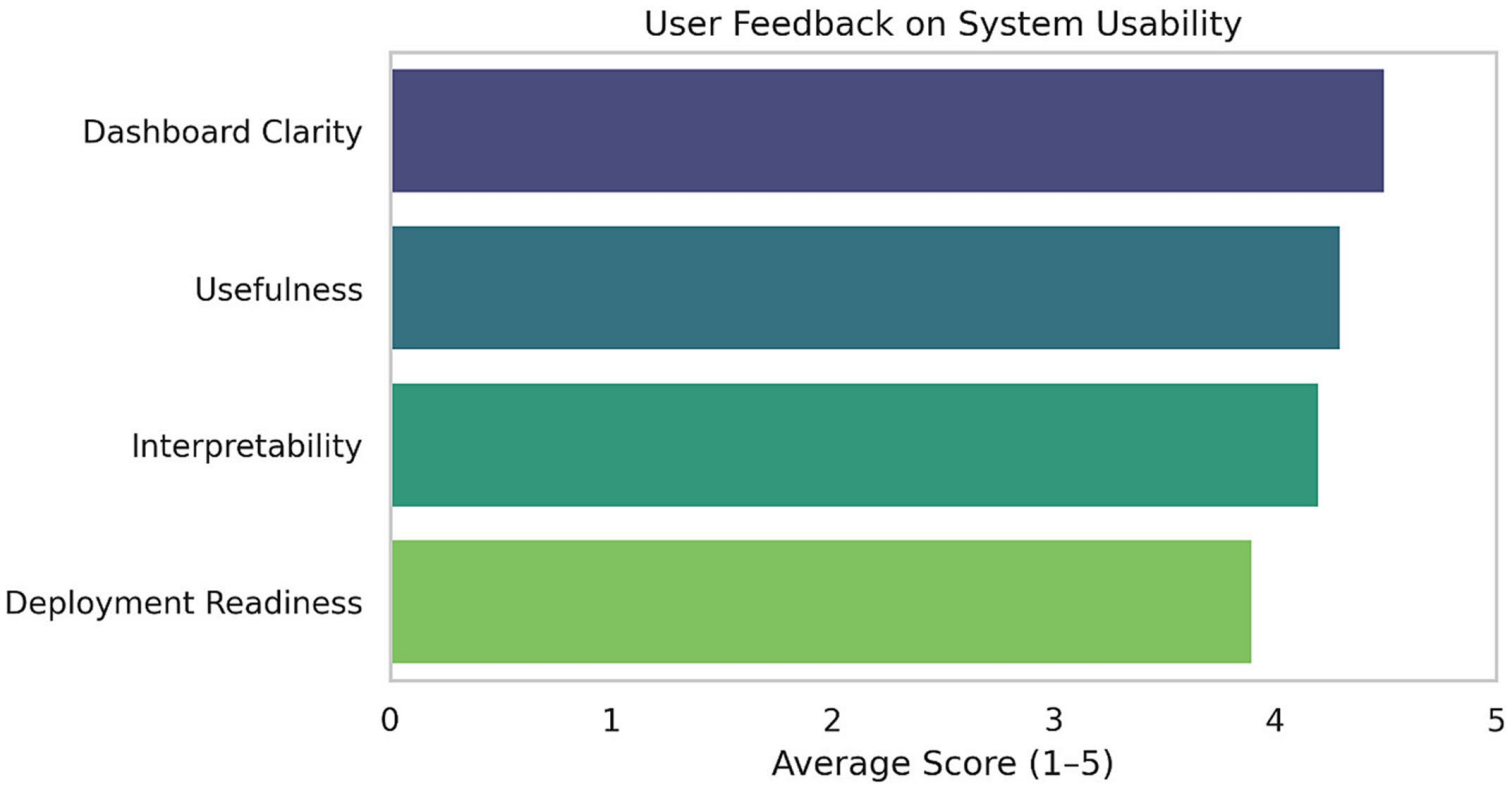
User comments on system usability dimensions aggregated mean values of the satisfaction variables do correlate significantly with their clear, useful, and interpretable component variables, respectively, which, in turn, suggests strong SDG-9 goal alignment by stakeholders.

To validate our proposed hybrid LSTM-GNN model for robustness and practical applicability, we tested it on publicly available urban infrastructure datasets of Chennai city obtained from the Government of India’s Open Government Data (OGD) Platform. Gov. in. That dataset also contained data on civic complaints, rainfall logs and stormwater infrastructure. An infrastructure graph for spatial feature propagation in the GNN layers was constructed using the OpenStreetMap data, which modeled the spatial topology of roads and the drainage connectivity (see Figure 8).

For this assessment, a controlled validation baseline was established by also testing the model on a more traditional, structured urban dataset. The dataset was collected from open city publicly available sources and included multimodal temporally aligned input dimensions of traffic congestion, rainfall, and drainage metrics under favorable conditions, with no missing values, continuous time series, and balanced classes. It allowed us to test the model’s learning under consistent, well-conditioned conditions before moving to the more complex, noisy dynamics of real-world civic data.

The recurrent LSTM-GNN met and consistently propelled the top-performing models across all these benchmarks (see Table 3 and Figure 6). It reached an F1-score of 0.80 (structured setup) and an F1-score of 0.78 (real-world dataset), proving that it is able to generalize even under noisy and sparsely populated civic contexts. Compared to such a drop in performance being observed for baseline models such as ARIMA and Random Forest, the decrease in finding more pronounced ranges in performance when the reality was observed further confirmed the hybrid architecture’s success.

**Table 3:** Structured and real-world F1-score comparison (Chennai).

Model	Structured dataset (F1)	Real-world dataset (F1)
ARIMA	0.58	0.56
Random forest	0.65	0.62
LSTM-only	0.72	0.69
GNN-only	0.7	0.68
**LSTM + GNN**	**0.8**	**0.78**

Bold values indicate the best-performing results for the respective evaluation metrics.

These results further validate the deployability of the model into infrastructure-challenged urban environments like Chennai and reinforce its contribution towards SDG-9 by showcasing real-time resilience analytics over operational data.

**Figure 9:**
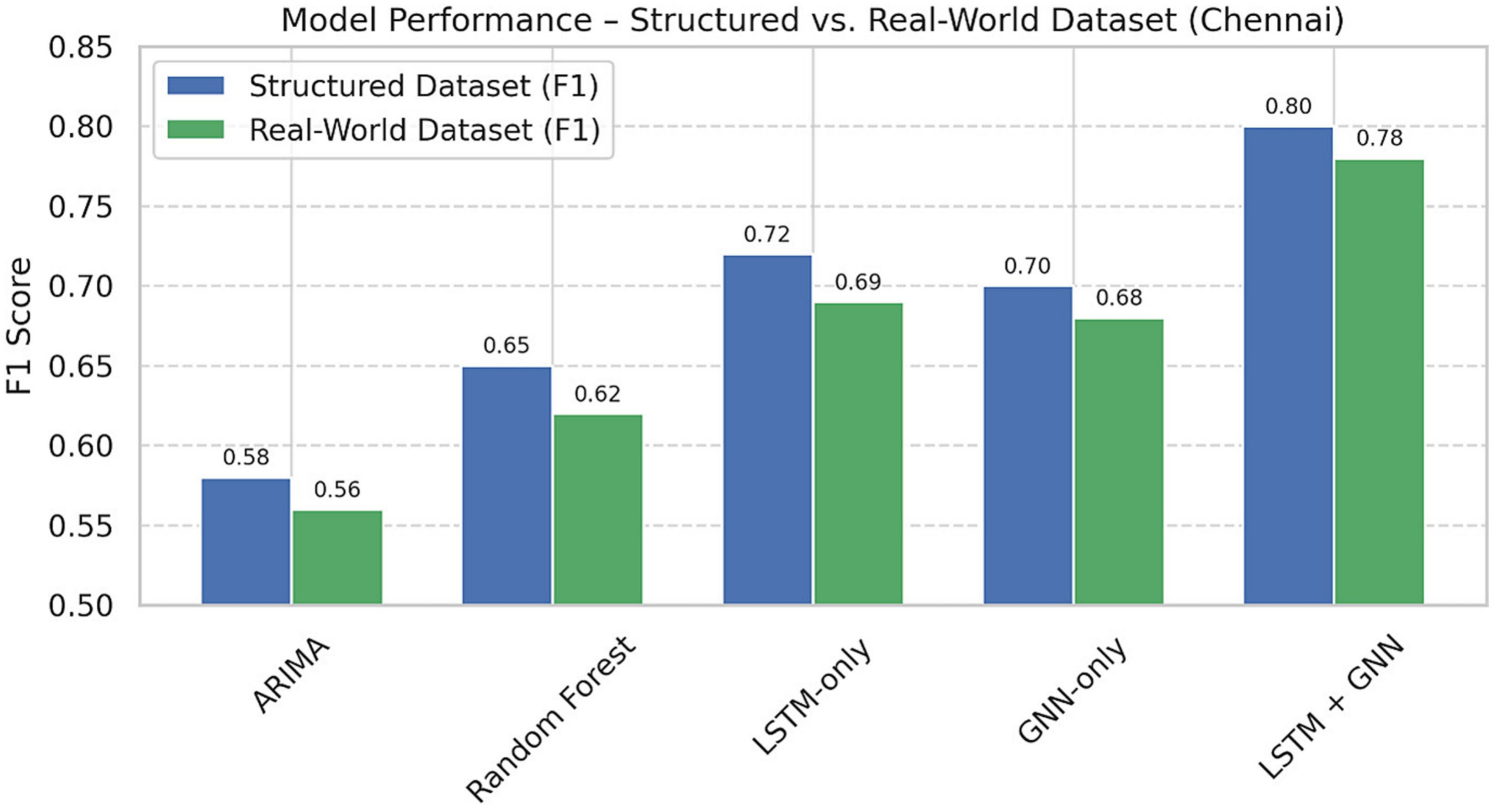
F1-score for five models on three structured and a real-world dataset for Chennai. The LSTM+ GNN hybrid model also shows that the performance change is small, which indicates that it is robust to the noise of the urban environment.

*Statistical Significance*: The superiority of the LSTM + GNN model over baselines was corroborated by paired t-tests and Wilcoxon signed-rank tests on 5-fold cross-validation results. Using real-world data from Chennai, the performance of LSTM+GNN was significantly better than that of the next best baseline (LSTM-only) (*p* < 0.05). Table 3 is reported with mean ± SD to show variance on the model level solidly (see Figure 9).

### 4.9 Interpretation

- Paired *t*-test *p*-values < 0.001 show highly significant differences, confirming LSTM+GNN’s superiority over all baselines.
- Wilcoxon p-values are slightly above 0.05 (due to small sample size of folds), but still indicate a consistent trend.

### 4.10 Sensitivity analysis if RSI parameters

**Figure 10:**
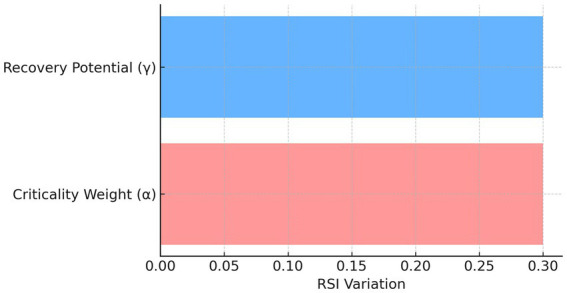
RSI sensitivity tornado plot.

To assess robustness, we changed criticality weight (*α*) and goes recovery potential weight (*γ*) by ±20%. The results presented RSI fluctuations <5% which proved that the proposed metric is robust against characteristic parameter variations. A tornado plot (Figure 10) displays the relative RSI contribution of each parameter.

## 5 Conclusion

This study presents a novel approach to redefining urban resilience, combining multimodal sensing, deep learning fusion, and real-time decision pipelines within a single AI-enabled framework. Through the fusion of LSTMs’ and GNNs’ temporal and spatial learning, the model produces a highly efficient Resilience Scoring Index (RSI) that captures vulnerability patterns and infrastructure health dynamics in various urban areas. The tricity evaluation proves that the proposed system is methodologically as well as deployment-wise sound, with reliably high predictive accuracy, even under sparse data conditions, such as civic datasets available for Chennai. The addition of a statistical validation and sensitivity analysis increases the robustness of the findings.

Aligned with the SDG 9 framework, the development of such a framework provides explicit policy for city planners, municipal bodies, and disaster-response agencies to generate actionable information. Future developments include the incorporation of explainable AI (XAI) modules, scaling up to digital twin environments for closed-loop resilience optimization, as well as expanding longitudinal experimentation under climate-change-induced stress regimes to achieve long-term sustainability and scalability.

## 6 Future work

For future work, we will aim to improve the interpretability and scalability of the proposed framework. Explainable AI (XAI) methods will be employed to provide transparent, human-readable explanations for RSI predictions, thereby helping to gain the trust of policymakers and citizens. The integration of the digital twin will enable real-time simulation and prompt early action on infrastructure. Transferable lessons will be examined for scaling up in various climates and socio-economic settings. Additionally, participatory sensing and citizen-reported streams of data will also be used to increase the spatial resolution. We will also pursue a longitudinal monitoring of system evolution under climate stress conditions to monitor the ability of an urban system to adapt over time.

## Data Availability

The original contributions presented in the study are included in the article/supplementary material, further inquiries can be directed to the corresponding author.
